# Dual-Channel Global Closed-Loop Supply Chain Network Optimization Based on Random Demand and Recovery Rate

**DOI:** 10.3390/ijerph17238768

**Published:** 2020-11-25

**Authors:** Aijun Liu, Yan Zhang, Senhao Luo, Jie Miao

**Affiliations:** Department of Management Engineering, School of Economics & Management, Xidian University, Xi’an 710071, China; ajliu@xidian.edu.cn (A.L.); shluo@stu.xidian.edu.cn (S.L.); jmiao@stu.xidian.edu.cn (J.M.)

**Keywords:** global closed-loop supply chain, dual-channel sales and dual-channel collection, mixed-integer linear programming, design and planning, ε-constraint method

## Abstract

In the process of globalization, customer demand is usually difficult to predict, and product recycling is generally difficult to achieve accurately. It is also urgent to deal with increased inventory while avoiding shortages, with the purpose of reducing supply chain risks. This study analyzes the integrated supply chain decision-making problem in the random product demand and return environment. It proposes a multi-objective optimization model, which is an effective tool to solve the design and planning problems of the global closed-loop supply chain. It consists of a multi-period, single-product and multi-objective mixed integer linear programming model, which can solve some strategic decision problems, including the network structure, entity capacities, flow of products and components, and collection levels, as well as the inventory levels. From the perspective of economic, environmental and social benefits, three objective functions are defined, including maximizing the net present value (NPV) of the system, minimizing the total $CO_{2e}$ emissions of supply chain activities, and maximizing social sustainability indicators. Finally, a numerical example is provided to verify the advantages of this model, and sensitivity analysis results are provided. The results show that changes in product demand and return rate will have a great impact on economic and social performance.

## 1. Introduction

Over the past decades, global supply chain (GSC) activity has grown tremendously as a result of decreased trade barriers and free trade agreements among countries. A GSC refers to the combination of supply chains on a global scale; it requires a global vision focused on extending the supply chain system to the whole world and selecting the most competitive partners around the world. Recent developments in the trade barrier debate and growing uncertainty about trade policies across countries are forcing companies to rethink their global operations. In addition, worldwide environmental and resource crises have become increasingly prominent in recent years, and global closed-loop supply chain management with the main characteristics of “recycling and remanufacturing” has attracted extensive attention from governments, companies and academia [1]. Many scholars have conducted extensive research on the issue from a global perspective, particularly the integrated optimization problem of the global closed-loop supply chain [2]. Alternately, with complicated commercial competition and the rapid development of the Internet, constantly trying to innovate business models is one of the ways for companies to succeed in fierce global competition, more and more companies establish online channels to sell products accompanied with their traditional retail channels. A recent survey reported that approximately 42% of the top-ranking companies sell their products to consumers through online sales channels in various industries [3,4]. Further, the dual-channel collection model has also become a measure for major companies. Compared with any traditional single-channel collection model, the dual-channel collection model can achieve better market penetration [5]. The combination of dual-channel sales and collection models (physical retail and collection channels + online sales and collection channels) is the main development trend of global closed-loop supply chains in recent years. The retailer’s collection strategies, as well as the manufacturer’s collection and remanufacturing strategies, have significant and complex influences on the operation and performance of the whole chain [6]. Therefore, it is of great theoretical and practical significance to study the integrated optimization problem of a dual-channel global closed-loop supply chain.

A GSC is a supply chain that transcends the borders of a single country. It mainly implements a series of interconnected business activities scattered around the world, including the purchase of raw materials and parts, the acquisition and processing of final products, activities to enhance product value-added, distribution, and information exchange between various commercial entities to reduce costs and expand revenue [7]. A GSC can achieve diversified customer docking and then help suppliers obtain relevant information and create knowledge through their close relationships with the customer base, thereby promoting product development [8]. Therefore, to achieve more effective competition, an increasing number of companies rely on relationships with GSC partners. A strong supply chain relationship can reveal unknown customer needs over time through in-depth interaction with customers and thus jointly create the relationship capital required to deepen knowledge [9]. However, GSCs are difficult to manage and control. In the context of globalization, larger geographic distances increase costs and complicate inventory decisions [10]. Of course, differences in culture, language, and conventions will also reduce the effectiveness of coordination with customers and suppliers to a certain extent [11]. Similarly, developing countries’ inadequate infrastructure in transportation and telecommunications, as well as backward equipment and technology, pose many challenges for developed countries to achieve global supply chains [12]. In addition, the GSC also has unique risks affecting performance, including the changes in the regulatory environment and the instability of factors such as costs, delivery, quality, and flexibility [13]. These risks are factored into consideration when designing global supply chains. Summarizing the previous research work of GSC, it usually takes multi-factory companies as the research object, focusing on enterprise decision-making models, and paying special attention to the connection between headquarters and branches. As an applied research, the GSC research work is guided by the current needs of multinational companies. It mainly answers the question: For multinational companies to conduct business activities in different regions, how do they make decisions to achieve optimality? That is, the multinational company decision problem. To help managers address these issues, the research community has developed many GSC design models. First of all, the decision of supplier selection has become the primary solution to global supply chain design problems [14]. In addition, the integration of decisions throughout the chain also affects the design of global supply chains [15]. Integrating business processes is an effective measure, mainly by integrating replenishment plans between companies by managing inventory and collaboration plans with suppliers, as well as sharing promotion information with replenishing companies. It also includes redesigning supply strategies, coordinating with suppliers, redistributing distribution strategies, and coordinating with customers. However, the benefits of GSCs may be hindered by the aforementioned improvement plans [16]. Therefore, this study aims to effectively design a GSC based on the relationships among the partners, and the optimal decision is to produce the maximum return. 

In recent years, research on the closed-loop supply chain has developed from the original closed-loop supply chain with single-channel sales and collection model to the current dual-channel closed-loop supply chain with dual-channel sales and collection model. To ensure the stable operation of the supply chain, many scholars and practitioners have conducted in-depth research on how to achieve closed-loop supply chain coordination. Among them, government subsidization [17,18], and relevant contracts and mechanisms [5,19] are considered as two direct and important measures to coordinate the supply chain. Government can encourage manufacturers to adopt the required channel structure by setting appropriate subsidy levels, the optimal price and the percentage of subsidy allocation will affect the final demand price of the remanufactured product. The customs contract and profit sharing contract can increase the profit of both online and offline channels of supply chain members by reasonably setting the proportion of revenue sharing and cost sharing, and finally achieve a win-win situation between dealers and collectors. In addition, other factors, such as service value [20], also affect the coordination and stability of the supply chain to a certain extent. Generally speaking, the causes of closed-loop supply chain coordination problems include information asymmetry, decentralized decision-making, high ambiguity, limited rationality, and socialist behavior. To solve these problems, we should seek solutions from the perspective of system, technology and culture, seeking information sharing, collaborative logistics and trust. Among them, the correct choice of sales and collection channel model is considered as one of the key measures to effectively coordinate the closed-loop supply chain [19]. In the forward network, a single sales channel where a retailer sells products directly to customers is a traditional sales model. Due to the rapid development of the Internet, e-commerce has gradually become popular, and manufacturers have also started retail businesses. Chen et al. [21] call the phenomenon of manufacturers selling products directly to customers through online channels as manufacturer encroachment, and they also found that manufacturer encroachment behavior can bring more profit to manufacturers than information asymmetry. Therefore, as e-commerce technology continues to mature, the dual-channel sales model, which combines offline retailer sales with online manufacturer sales, has gradually become a measure taken by major companies. In the reverse network, there are three common types of collection models implemented by companies for returned products, including retailer, manufacturer and third-party collection. Wan and Hong [22] build a game model to analyze the optimal pricing and collection policies for retailers and third-party collection. There is no doubt that dual-channel collection will cause the existence of a competitive relationship between the collecting entities, which will affect the profit of the collecting entities to a certain extent. Zheng et al. [23] point out that collection competition will reduce the amount of total collection and the expected profit for all supply chain members. The expected profits can only be improved if the two collection agencies can still carry out intensive collection work without considering the reward and punishment mechanism. Liu et al. [16] also believe that there is collection competition between dual collection channels, to choose the most appropriate dual-channel collection model, they study the closed-loop supply chain reverse channel selection decision problems in a pairwise combination of the three collection modes. It is found that the manufacturer and retailer dual collection model is the best choice regardless of the intensity of competition. Later, Modak et al. [24] confirm this conclusion by analyzing and comparing the above three collection models for second-hand products. It turns out that third party participation in second-hand product collection is always detrimental. In addition, some rare collection models, such as collector collection [25] and backup supplier collection [26], have not been popularized due to fewer businesses using them. 

Supply chain network design is one of the most critical planning problems in supply chain management. Network design helps the supply chain to determine the location of production-, storage-, and transportation-related facilities, as well as the capacity and functionality of the equipment, which are critical for the long-term operation of the supply chain. For a general supply chain network, according to its characteristics, the supply chain can be categorized as a green, sustainable, risky, resilient, or uncertain supply chain. The implementation plan and goals of its network design vary according to its characteristics. Rezaee et al. [27] propose a planning model to design green supply chains in a carbon environment. Varsei and Polyakovskiy [28] build a supply chain analysis model to solve wine supply chain network design issues considering three aspects of sustainability. Babazadeh et al. [29] propose a model to design a biodiesel supply chain network at risk. Rezapour et al. [30] design a nonlinear model to solve the design problem of resilient automobile supply chain networks, they use three strategies to reduce the risk of outages. Zokaee et al. [10] build a model for the supply chain design based on the uncertainty, to determine the strategic "location" and tactical "allocation" decisions of the supply chain. Compared with the general supply chain, the closed-loop supply chain reduces the pollution emissions and surplus waste by closing the flow of materials, while providing services to customers at a lower cost, its network design is also gradually being valued. For the single objective optimization model, minimizing costs is generally considered the primary goal of designing closed-loop supply chains [31,32,33]. Li et al. [31] establish a nonlinear model to study the inventory decision problems of closed-loop systems with third-party logistics services, it aims to determine the location of the mixed distribution collection center while minimizing the total cost of the system. Rad et al. [32] construct a mathematical model, in which multi-period, multi-product and single-objective characteristics are taken into account; it is mainly used to determine the production plan, inventory level, flow between facilities, transportation type, procurement quantity, etc., by minimizing the cost of the system. For the multi-objective optimization model, environmental impact is generally considered as the second objective function, and the environmental impact is usually measured by the carbon dioxide emissions and the green performance of the system [34,35,36]. Chen et al. [34] develop a deterministic model to solve the problem of the multi-objective design for the solar industry; the single-period, single-product and multi-objective model is provided to find the balance between total costs and total carbon dioxide emissions, as well as determine the factory location selection, capacity expansion, technology selection, and purchase and order fulfillment decision problems. Tosarkani and Amin [35] extend the original multi-period, multi-product and single-objective optimization model and consider the green performance related to the collection center. The objective is to maximize the green factors of the system on the basis of maximizing the profit of the supply chain. Other objective functions, such as sales loss [37] and social performance [38], can also be optimized according to the actual research problems. The acceleration of economic globalization has led to the emergence of GSCs. Due to the complexity of GSCs, only some scholars have conducted research related to network design. Hasani and Khosrojerdi [11] develop a mixed integer nonlinear model for designing GSC networks under uncertainty. They propose six flexible strategies to mitigate the risks of related outages to extract management insights. Urata et al. [13] propose an Asian GSC network design method, which can minimize material-based carbon dioxide emissions costs and determine suppliers, plant locations, and databases that meet low-carbon material supply needs. Amin and Baki [12] consider global factors such as exchange rates and tariffs, and propose a GSC network design model based on uncertain demand. Due to the difficulty of managing and controlling GSCs, research on the network design of these supply chains is not very mature, especially in terms of the design and optimization of dual-channel global closed-loop supply chains.

In order to better solve the closed-loop supply chain network design model, the ε-constraint method is used in this paper. The ε-constraint method can generate all effective solutions to the problem, and the decision maker selects the most suitable one from them. Many studies have verified the effectiveness of this method. Talaei et al. [39] apply the ε-constraint method to solve the bi-objective optimization model. They list three advantages of the method. First, with the change of ε, the model can get different optimal solutions. Second, the scale of objective function does not affect the optimal solution. Third, this method is very suitable for solving non-convex models. Jindal and Sangwan [36] use the interactive ε-constraint method to solve the proposed multi-objective optimization model. They think that since the decision-makers only participate in the second stage of the solution, this method can have a good decision-making effect when the decision-maker interaction is difficult. Yang et al. [40] put forward an improved ε-constraint method to solve the model. The improved method can adjust the value of ε according to the size of infeasible individuals, which can effectively prevent the unreasonable setting of ε value. At the same time, it can switch freely to participate in a global and local search, which can balance the convergence. Shafiekhani et al. [41] propose the augmented ε-constraint method to solve the bi-objective optimization problem. The augmented ε-constraint method can avoid the emergence of various weak solutions due to changing the value of ε, and reduce the repetition of the solution process to accelerate the whole process. Tabar et al. [42] also use the augmented ε-constraint method to solve the proposed optimization model. They think that the augmented ε-constraint method does not change the original feasible region in the process of solving, and can produce non inferior solutions. At the same time, the scaling of the objective function does not affect the process and result of the solution. Osorio et al. [43] propose an augmented ε-constraint method combined the with sample average approximation method to solve linear programming model. Each ε can be used to solve a sample average approximation problem. The resulting Pareto front can be composed of the assignment of one target and the expected value of another. This method can avoid the emergence of weak solutions and accelerate the whole process of solving. Dorotić et al. [44] combine the ε-constraint method with the weighted sum method for the multi-objective optimization problem, and then they use the inflection point method to select the solution closest to the Pareto optimal solution from the effective solutions. This method can realize the visualization of the Pareto front and obtain the compromise solution based on all possible results. Fan et al. [45] propose an improved ε-constraint processing method, which is combined with a multi-objective evolutionary algorithm to solve optimization problems. The integration method can dynamically adjust the value of ε according to the ratio of feasible to total solutions, so as to ensure the balance between feasible and infeasible regions. 

The integrated optimization is one of the fundamental activities and infrastructure construction for supply chain management, which affects the operational efficiency. In addition, product collection helps reduce production costs, reduce energy consumption, and protect the environment, the optimization of a global closed-loop supply chain with dual-channels can effectively control the operation of the forward and reverse supply networks, as well as promote a close relationship between product sales and product collection. Therefore, this paper establishes a single-product, multi-period, multi-objective mixed-integer linear programming model, and a dual-channel sales and dual-channel collection model are designed. The model integrates three conflicting objective functions: the economic factors are measured by net present value (NPV), the environmental impact is calculated by the total amount of $CO_{2e}$ emissions, and social welfare is measured by the social sustainability indicator. In addition, the optimization of the objective functions can solve the strategic decision problem, including the determination of the network structure, entity capacity, the flow of products and components between entities, returned product collection levels, and production, remanufacturing and inventory levels. Finally, a proposed solution method, i.e., the ε-constraint method, is provided, in which three different size test problems are assessed. The result verifies the applicability and robustness of the solution method and provides management advice for other supply chain managers.

## 2. Materials and Methods 

### 2.1. Problem Definition

**Figure 1:**
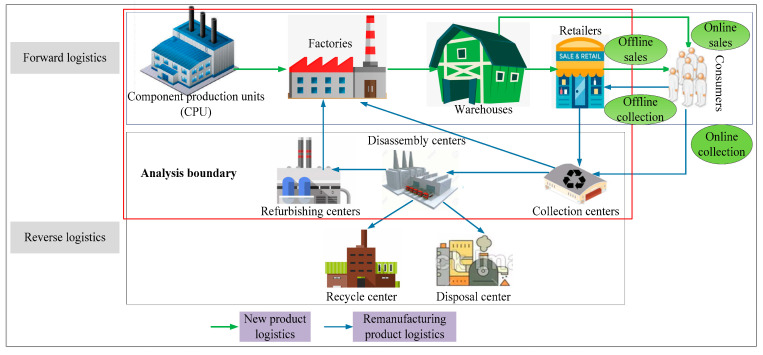
Dual-channel network structure.

On the basis of a four-echelon network framework studied by Mota et al. [38], this paper refines a dual-channel network structure constituting a five-echelon structure in the forward supply chain and a five-echelon structure in the reverse supply chain. Meanwhile, collection, disassembly, refurbishing, recycle and disposal center are integrated into the existing closed-loop network, and the reverse logistics operation process is studied in more detail. The provided network structure is shown in Figure 1. This network includes a supply chain with dual-channel sales and dual-channel collection, consisting of new product logistics and remanufacturing product logistics stages. The supply chain contains ten supply chain entities, namely, the component production unit (CPU), factory, warehouse, retailer, consumer, and collection, disassembly, refurbishing, recycle, disposal center. In the forward network, components are supplied to the factories by the CPUs, where they can be manufactured into final products by using a variety of production machines. Then, the final products are sent to the warehouses, and the excess products remain in the warehouses as inventory after satisfying the needs of the retailers and consumers; thus, a dual-channel supply model is employed. On the one hand, manufacturers provide products directly to consumers through electronic direct sales channels. On the other hand, retailers act as a medium between the warehouses and consumers, and manufacturers sell products to consumers through offline stores, which are referred to as retailers. In the reverse supply chain stage, a dual-channel collection model is employed, and there are two ways for manufacturers to take responsibility for product collection. One is to entrust retailers to take responsibility for collection, and the retailers are also responsible for the collection of returned products when they sell new products; then, the products collected by the retailers are shipped to the collection centers. Using the other approach, collection centers collect the returned products from consumers by online product collection. In collection centers, the collected products are inspected, tested, and classified according to the length of use and the degree of oldness/newness. Some of the newer products are sent to the factories for further circulation as new products after minor repairs. The remaining products are sent to the disassembly centers, which are responsible for dismantling the products to obtain different components. The disassembled components can be sent to three places, namely, recycle centers, refurbishing centers and disposal centers, based on whether the components are still functional. The refurbished components that are considered to be new will participate in the remanufacturing process of the factories. The factory’s demand for components is also met by the CPUs; the recycled components can garner a certain amount of recycling profit for the recycler, and the disposed components are considered useless. In real life, not all products can be collected by the collection centers; the collected products represent only a portion of all the previously sold products.

To verify the applicability of the proposed network structure, a numerical example considering multiple entities, multiple periods and multiple objectives is proposed. The multi-objective mathematical model can be used to determine the following decisions:

(1) The dual-channel global closed-loop supply chain network structure.
(2) The capacity of each entity in the network structure.
(3) The flow of components and products between entities.
(4) The collection level of returned products.
(5) The production, remanufacturing and inventory levels of products.

They are determined by integrating three objective functions:

(1) Maximize the economic factors of the system, measured by the NPV over the time range.
(2) Minimize the environmental impact of the system, measured by the $CO_{2e}$ emissions amount over the time range.
(3) Maximize the social benefits of the system, measured by the social sustainability indicator proposed in this paper. It is related to the four criteria presented, which reflect the social benefits of the system for social sustainable development.

### 2.2. Assumptions

- The cost of remanufacturing a product is lower than the cost of manufacturing a new product.
- Multiple time periods and a single product are considered.
- Logistics activities in the forward and reverse supply chain stages are completed by the company’s internal transportation fleet.
- The factories are responsible for the production of new products and the remanufacturing of returned products and refurbished components, as well as ensuring that the new products and remanufactured products are of the same quality.
- The disposal center will charge a disposal fee for the components that are discarded during the remanufacturing process.
- The quantity of the collected products is less than the quantity required by the market, so when the collected products are used to produce the remanufactured products, it is also necessary to produce new products.
- All returned products are collected, and all demands are met.

### 2.3. Notations

Based on the notations used in previous studies, this paper extends them, taking into account the multiple cycles, and focusing on the research gaps on the influence of the quality level for returned products on the collection of components, in the remanufacturing process. Finally, the following notation collection is proposed.

The sets used in the model are provided in Table 1.

The parameters are classified as supply and capacity-related, cost, revenue and price-related, $CO_{2e}$ emissions amount-related, distance-related, etc., and are provided in Appendix A.

The decision variables are provided in Table 2.

### 2.4. Objective Functions

The three conflicting objective functions are modeled as follows: (1) maximizing the economic factors of the dual-channel global closed-loop supply chain, (2) minimizing the environmental impact of all supply chain activities, (3) maximizing the social benefits of the entire global closed-loop system.

Objective 1 (economic): Maximizing the economic factors.

Given that Mota et al. [38] used NPV as an indicator to measure the economic performance, in this study, the NPV is maximized to construct the economic objective function.

Because here, a certain time range of the cash flow is studied, the time range can be disassembled into several time periods for further research. The original investment includes the original investment of the entities, the original investment in the production machines, and the original investment in truck transport. Therefore, maximizing the NPV can be expressed as Equation (1).

(1) $$\mathrm{Max}NPV=\sum_{t}\frac{G_{t}}{{\left(1+R_{0}\right)}^{t}}-\left(\sum_{m}F_{m}+\sum_{j}F_{j}+F_{0}\right)$$

In addition, the cash flow in time period *t* is quantified by Equation (2). In any time period before the last time period, the cash flow is equal to the net profit in the time period, and in the last time period, the cash flow is equal to the net profit in that time period plus the residual value of the original investment.

(2) $$G_{t}=\{\begin{array}{c} H_{t},t=1,2,\ldots ,Q_{0}-1\\ H_{t}+\left(\sum_{m}T_{m}F_{m}+\sum_{j}T_{j}F_{j}+T_{0}F_{0}\right),t=Q_{0} \end{array}$$

The sources of revenue include the warehouses selling new products to consumers, the retailers selling new products to consumers, and the disassembly centers selling discarded components to the recycle center. Equation (3) represents the total revenue in time period *t*.

(3) $$I_{t}=\sum_{d}\sum_{f}C_{2t}C_{dft}^{0}+\sum_{e}\sum_{f}C_{1t}C_{eft}^{0}+\sum_{a}\sum_{h}B_{a2}F_{aht}^{0}$$

To determine the profit in time period *t*, all costs incurred in time period *t* must be determined. These costs include the cost of producing components in CPUs (term 1); the cost of using production machines (electricity costs, equipment maintenance costs, equipment maintenance costs, etc.), including the cost of using the production machines for production (term 2); the costs associated with remanufacturing (term 3) and minor repairs (term 4) of products; the cost of collecting returned products, including the cost for collecting returned products from consumers to collection centers (term 5) and retailers (term 6); the cost of truck transport (truck fuel costs, maintenance costs, etc.) for components shipped from the CPUs to factories (term 7); the cost of truck transport for product circulation, including the cost for products shipped from factories to warehouses (term 8) and the cost for products shipped from warehouses to retailers (term 9) and consumers (term 10); the cost of truck transport for returned product collection, including the cost for returned products shipped from consumers (term 11) and retailers (term 12) to collection centers; the cost of truck transport for returned products shipped from collection centers to factories (term 13) and disassembly centers (term 14); the cost of truck transport for components shipped from disassembly centers to refurbishing centers (term 15), recycle centers (term 16) and disposal centers (term 17); the cost of truck transport for components shipped from refurbishing centers to factories (term 18); the inventory holding cost for warehouses (term 19); the cost of disassembling returned products at disassembly centers (term 20); the cost of refurbishing components at refurbishing centers (term 21); the cost of disposing of components at disposal centers (term 22); the labour cost of workers at the entities, including the labour cost incurred at the CPUs (term 23), factories (term 24), warehouses (term 25), retailers (term 26), collection centers (term 27), disassembly centers (term 28) and refurbishing centers (term 29); the labour cost of employees working with production machines (term 30); and the rental cost of retailers in time period *t* (term 31). Equation (4) represents the total cost in time period *t.*

(4) $$\begin{array}{l} J_{t}=\sum_{a}\sum_{b}\sum_{c}B_{ab}B_{abct}^{0}+(\sum_{j}\sum_{c}C_{j}K_{jct}^{0}+\sum_{j}\sum_{c}C_{j}L_{jct}^{0}+\sum_{j}\sum_{c}C_{j}J_{jct}^{0})\\ +(\sum_{k}\sum_{f}\sum_{g}C_{kfg}D_{kfgt}^{0}+\sum_{k}\sum_{f}\sum_{e}C_{kfe}D_{kfet}^{0})+\sum_{a}\sum_{b}\sum_{c}D_{abc}M_{bc}B_{abct}^{0}\\ +(\sum_{c}\sum_{d}D_{cd}M_{cd}B_{cdt}^{0}+\sum_{d}\sum_{e}D_{de}M_{de}C_{det}^{0}+\sum_{d}\sum_{f}D_{df}M_{df}C_{dft}^{0})\\ +(\sum_{f}\sum_{g}D_{fg}M_{fg}D_{fgt}^{0}+\sum_{e}\sum_{g}D_{eg}M_{eg}D_{egt}^{0})+(\sum_{g}\sum_{c}D_{gc}M_{gc}E_{gct}^{0}\\ +\sum_{g}\sum_{h}D_{gh}M_{gh}E_{ght}^{0})+(\sum_{a}\sum_{h}\sum_{i}D_{ahi}M_{hi}F_{ahit}^{0}+\sum_{a}\sum_{h}D_{ah1}M_{h1}F_{aht}^{0}+\sum_{a}\sum_{h}D_{ah2}M_{h2}G_{aht}^{0})\\ +\sum_{a}\sum_{i}\sum_{c}D_{aic}M_{ic}G_{aict}^{0}+\sum_{d}B_{dt}H_{dt}^{0}+\sum_{g}\sum_{h}B_{0}E_{ght}^{0}+\sum_{a}\sum_{h}\sum_{i}B_{a1}F_{ahit}^{0}\\ +\sum_{a}\sum_{h}B_{a3}G_{aht}^{0}+(\sum_{b}Q_{b}P_{1}Q_{t}+\sum_{c}Q_{c}P_{2}Q_{t}+\sum_{d}Q_{d}P_{3}Q_{t}\\ +\sum_{e}Q_{e}P_{4}Q_{t}+\sum_{g}Q_{g}P_{5}Q_{t}+\sum_{h}Q_{h}P_{6}Q_{t}+\sum_{i}Q_{i}P_{7}Q_{t})\\ +\sum_{j}O_{j}P_{2}Q_{t}+\sum_{e}F_{et}A_{e}^{0} \end{array}$$

Therefore, the total profit in time period *t* can be expressed by $I_{t}-J_{t}$, and the net profit in time period *t* is shown as follows.

(5) $$H_{t}=(1-S_{0})(I_{t}-J_{t})$$

Objective 2 (environment): Minimizing the environmental impact.

Govindan et al. [46] pointed out that $CO_{2e}$ is one of the main substances that has a serious impact on the ecology and nature of the earth. A large amount of $CO_{2e}$ emissions not only pollutes the environment but also leads to irreversible and serious harm to the earth. Therefore, in this paper, the total amount of $CO_{2e}$ emissions generated by the entire supply chain activity is calculated based on the amount of $CO_{2e}$ emitted by different supply chain activities, thereby measuring the environmental impact.

The total $CO_{2e}$ emissions consist of four parts: (1) $CO_{2e}$ emissions generated by the construction of entities. (2) $CO_{2e}$ emissions produced by truck transport. (3) $CO_{2e}$ emissions derived from the production, remanufacturing and minor repairs of products. (4) $CO_{2e}$ emissions produced by producing components, disassembling products, refurbishing components and disposing of components. Thus, Equation (6) describes the total amount of $CO_{2e}$ emissions in the time range.

(6) $$\begin{array}{l} \mathrm{Min}CO_{2e}=(\sum_{b}L_{b}A_{b}^{0}+\sum_{c}L_{c}A_{c}^{0}+\sum_{d}L_{d}A_{d}^{0}+\sum_{g}L_{g}A_{g}^{0}+\sum_{h}L_{h}A_{h}^{0}+\sum_{i}L_{i}A_{i}^{0})\\ +2L_{0}(\sum_{b}\sum_{c}M_{bc}+\sum_{c}\sum_{d}M_{cd}+\sum_{d}\sum_{e}M_{de}+\sum_{d}\sum_{f}M_{df}+\sum_{e}\sum_{g}M_{eg}+\sum_{f}\sum_{g}M_{fg}+\sum_{g}\sum_{c}M_{gc}\\ +\sum_{g}\sum_{h}M_{gh}+\sum_{h}\sum_{i}M_{hi}+\sum_{h}M_{h1}+\sum_{h}M_{h2}+\sum_{i}\sum_{c}M_{ic})+(\sum_{c}\sum_{t}L_{1}K_{ct}^{0}+\sum_{c}\sum_{t}L_{2}K_{ct}^{0}\\ +\sum_{c}\sum_{t}L_{3}J_{ct}^{0})+(\sum_{a}\sum_{b}\sum_{c}\sum_{t}L_{a}B_{abct}^{0}+\sum_{g}\sum_{h}\sum_{t}L_{4}E_{ght}^{0}+\sum_{a}\sum_{h}\sum_{i}\sum_{t}L_{a1}F_{ahit}^{0}+\sum_{a}\sum_{h}\sum_{t}L_{a2}G_{aht}^{0}) \end{array}$$

Objective 3 (society): Maximizing the social benefits.

For manufacturing and remanufacturing processes, four criteria are selected to measure the social effects, namely, social sustainability features. The criteria chosen share certain commonalities, i.e., all of the criteria are moving towards the goal of achieving social sustainable development, and ensure that the differentiation in the manufacturing and remanufacturing processes is reflected. The four selected criteria and the related descriptions are shown in Table 3. Here, the interdependencies between the criteria are ignored, so the weights can be calculated by the analytic hierarchy process (AHP), and the matrix for the criteria and the goal (social sustainability) is shown in Table 4. The total impact is expressed as Equation (7).

(7) $$\mathrm{Max}K_{0}=\sum_{g}\sum_{h}\sum_{i}w_{1}K_{1}A_{g}^{0}A_{h}^{0}A_{i}^{0}+\sum_{g}\sum_{h}\sum_{i}w_{2}K_{2}A_{g}^{0}A_{h}^{0}A_{i}^{0}+w_{3}K_{3}+w_{4}K_{4}$$

### 2.5. Constraints

The constraints mainly include demand constraints, flow balance constraints, entity balance constraints, production machine balance constraints, supply capacity constraints, entity capacity constraints, flow capacity constraints, impact index constraints, binary variable constraints and nonnegative constraints. These constraints are provided in Appendix B. 

### 2.6. ε-Constraint Method

The exact or heuristic method can be used to determine the Pareto solutions. Among them, the threshold optimization method and the ε-constraint method are widely used. The principle of the threshold optimization method is to use the difference between the target and the problem background in gray scale, and divide the optimal level into several categories by setting the threshold, so as to realize the separation of the target and the problem background. The threshold optimization method is simple to calculate, but the determination of the optimal threshold is more complicated. The effect of determining the threshold by the threshold principle is not ideal, and the information distortion is more serious. The core idea of the ε-constraint method is to manually set the value of ε, and divide it into different regions based on the individual’s constraint default degree. In different regions, different evaluation methods are used to solve feasible and infeasible solutions. This method uses the information of infeasible solutions with better objective function values in the infeasible region, and has better convergence performance. Therefore, an exact method, the ε-constraint method, is applied. The ε-constraint method is actually a method that generates all of the Pareto optimal solutions or dominating solutions to the problem, and then, the decision-maker selects the best solution from all solutions based on the research goal.

According to the ε-constraint method, the multi-objective optimization problem expressed in Equation (8) can be transformed into the single-objective optimization problem expressed in Equation (9) by setting the appropriate ε.

(8) $$\begin{array}{l} \mathrm{Min}(Z_{1}(x),Z_{2}(x),\ldots ,Z_{\gamma }(x))\\ s.t.\\ x\in \chi \end{array}$$

where $Z_{1}(x)$, $Z_{1}(x)$, …, $Z_{\gamma }(x)$ are γ objective functions, x refers to the vector of decision variables, and χ represents the space of the solutions.

(9) $$\begin{array}{l} \mathrm{Min}Z_{1}(x)\\ s.t.\\ Z_{2}(x)\leq \varepsilon_{2}\\ Z_{3}(x)\leq \varepsilon_{3}\\ \ldots \\ Z_{\gamma }(x)\leq \varepsilon_{\gamma }\\ x\in \chi \end{array}$$

Therefore, for the provided problem, the ε-constraint model is expressed as:

(10) $$\begin{array}{l} \mathrm{Max}NPV\\ s.t.\\ CO_{2e}\leq \varepsilon_{2}\\ K_{0}\geq \varepsilon_{3}\\ \left(A1)-(A33\right) \end{array}$$

Then, to estimate the range of values of $\varepsilon_{2}$ and $\varepsilon_{3}$, the second and third objective functions are optimized separately under the defined constraints to obtain the corresponding result, which can be expressed as Equations (11) and (12).

(11) $$\begin{array}{l} \mathrm{Min}CO_{2e}\\ s.t.\\ \left(A1)-(A33\right) \end{array}$$

(12) $$\begin{array}{l} \mathrm{Max}K_{0}\\ s.t.\\ \left(A1)-(A33\right) \end{array}$$

Finally, the values of $\varepsilon_{2}$ and $\varepsilon_{3}$ are set.

Pareto optimality refers to a state of resource allocation that does not make anyone’s situation worse, and it is impossible to make some people’s situation better. The Pareto optimal solution set refers to a set of solutions that satisfy such conditions. In the practical application problem, when the ε-constraint method is applied, the Pareto optimal solution set with finite number of points is often obtained, but it is not known whether the solution set is the theoretical Pareto optimal solution set. This raises an important question as to whether the ε-constraint method can converge to the real Pareto front end. Relevant explanations and proofs are provided in Appendix C.

## 3. Results

To verify the effectiveness and practicability of the method so that companies of all sizes can be considered, three differently scaled dual-channel models are explored using the proposed ε-constraint method. Assume that the model can be expanded to include up to three CPUs (C1, C2, C3), four factories (F1, F2, F3, F4), two warehouses (W1, W2), four retailers (R1, R2, R3, R4), four consumers (O1, O2), three collection centers (L1, L2, L3), two disassembly centers (D1, D2), and four refurbishing centers (E1, E2, E3, E4). The sizes of the test problems assumed are shown in Table 5.

**Figure 2:**
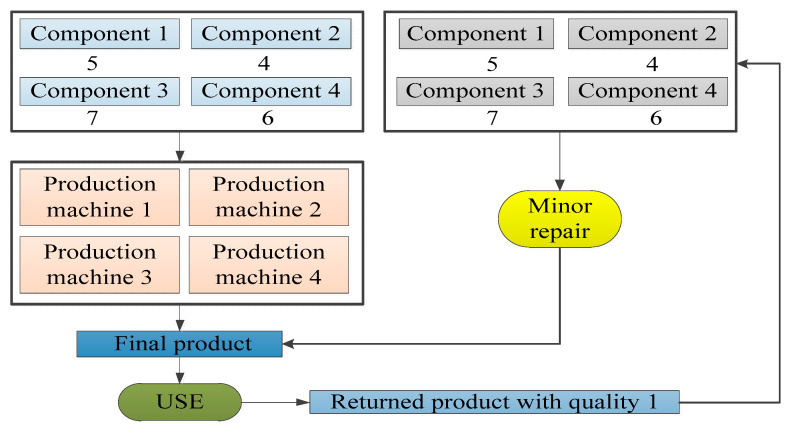
The relation of components, product machines, products and returned products with quality 1.

**Figure 3:**
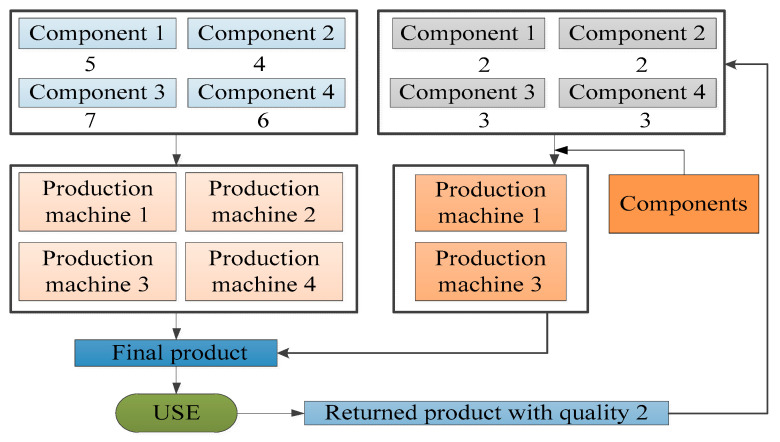
The relation of components, product machines, products and returned products with quality 2.

**Figure 4:**
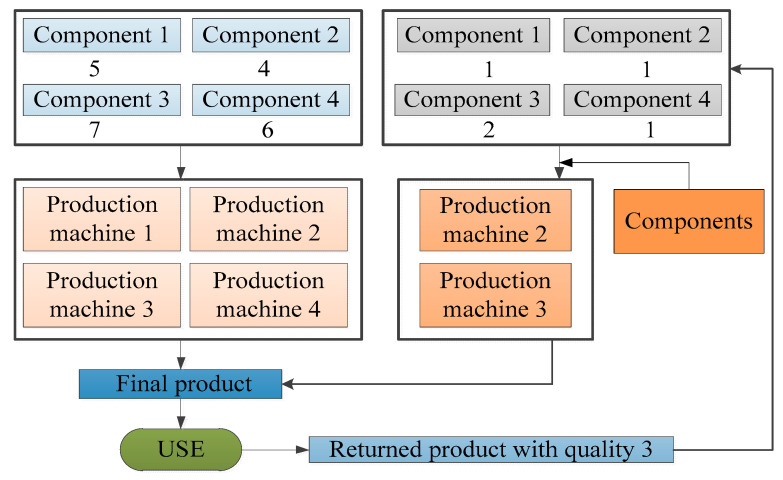
The relation of components, product machines, products and returned products with quality 3.

Suppose that a company produces and sells one type of product (FP), which is produced from four types of components (M1, M2, M3, M4), including five units of M1, four units of M2, seven units of M3 and six units of M4 and that the production process needs to be completed on four production machines (P1, P2, P3, P4). Then, the products will be collected after use, and the returned products can be divided into three different levels of quality (RP1, RP2, RP3), where a returned product with quality 1 requires only minor repairs without the need to use production machines and the remaining returned products will be disassembled into various components. One unit of returned product with quality 2 can be disassembled to obtain two units of M1, two units of M2, three units of M3 and three units of M4, and one unit of returned product with quality 3 can be disassembled to obtain one unit of M1, one unit of M2, two units of M3 and one unit of M4. Finally, by replenishing the missing components, one unit of the remanufactured product can be obtained, the minor repair process for returned products with quality 1 can be completed without replenishing components, the production process for returned products with quality 2 can be completed by P1 and P3, and the remanufacturing process for the returned products with quality 3 can be completed by P2 and P3. These processes are shown in Figure 2, Figure 3 and Figure 4. Table 6 describes the composition of products, and the demand for the machines used for producing and remanufacturing products. 

Table 7 describes the capacity characteristics of entities within the analysis boundary, including the maximum and minimum supply capacity of the CPUs, as well as the maximum receiving capacity for the components, products and returned products of other entities. Table 8 depicts the cost, revenue and proportion characteristics associated with the components, the production, the refurbishing, recycling, disposal and transportation costs, and the related proportions of the components. Table 9 depicts the cost characteristics associated with the final products and returned products, including the cost of disassembly and collection for the returned products, as well as the production machine operating cost and transportation cost for the products and returned products. Table 10 lists the construction and worker characteristics associated with the entities within the analysis boundary, including the entity construction costs, amount of $CO_{2e}$ emissions generated during entity construction, number of workers required, and wages of workers. Then, the distance between entities is shown in Table 11. Next, Table 12 provides the amount of $CO_{2e}$ emissions associated with supply chain activities, including production, minor repairs, remanufacturing, disassembly, refurbishing, and disposal. 

Although the size of the test problem is constantly increasing, the parameter values of the three test problems are fixed, which allows for an evaluation of the influence of the proposed method on differently scaled models. In addition, a test problem with a time period of one year, i.e., 52 weeks, is studied, and the total time range is three years. 

Next, some time-related parameters are introduced. For this type of product, the inventory holding cost in the warehouse changes with time. In the three years studied, the inventory holding cost per unit product is $5, $6 and $7, respectively. Due to the upgrading of products and maturing production technology, the unit selling price of the product decreases with time and is $500, $480 and $450, respectively, which is consistent with the selling price of the retailers and warehouses. In addition, the cost of leasing increases year by year, at $10,000, $12,000 and $13,000, respectively, per year for a store. The demand for the product also increases year by year in the three years, reaching 1000, 1200 and 1400, respectively. More importantly, due to the increased awareness of the public regarding energy conservation in recent years, a large number of returned products are collected to complete the remanufacturing process of new products, and the return rate of the product reaches 30%.

In each factory, four production machines are used to complete the production and remanufacturing activities, and mass production of the products is carried out. The cost of acquisition for four production machines varies according to the tasks they perform and their effects: $7000, $6000, $9000 and $12,000. For these four production machines, six, nine, five and seven workers are required to complete the manufacturing activities during the operation. At the same time, to facilitate transportation and ensure the timeliness of the arrival of the components and products, the company has set up its own transport fleet to complete all transport activities, replacing the original third-party logistics provider’s transportation model, and the transport personnel are transport workers at the transport entity. At present, to make the number of trucks sufficient to meet the existing traffic volume and not waste resources, the number of trucks, depending on the size of the problem being studied, is 8, 14 and 16, and the cost of each truck is $80,000. There is no doubt that trucking will produce harmful substances that pollute the environment. Here, only $CO_{2e}$ emissions are considered. For a transport distance of 1 km, the $CO_{2e}$ emissions per truck are 400 g. For production machines and trucks, the residual value will decrease with an increase in the service life. At the end of the three years studied, the residual ratio of the four machines is 35%, 38%, 30% and 41%, and the residual ratio of these trucks is 35%. For entities within the analysis boundary, the residual ratio varies with the type of entity, and the ratios are 44%, 42%, 48%, 38%, 36% and 35%.

The regional index values of extended producer responsibilities and employment practices are considered to be location-dependent on the hybrid collection facility and subject to the size of the test problem. Therefore, the index values of extended producer responsibilities for the three differently sized test problems are 0.8, 0.85 and 0.92, and the index values of the employment practices for the three differently sized test problems are 0.6, 0.73, 0.9. In addition, the weights of the four social sustainability criteria, which are used to calculate the social benefits, can be calculated through AHP, where the pairwise comparison matrix of the social sustainability goal and criteria is obtained by surveying and consulting different manufacturers in the study area. Therefore, the relative weights of the four criteria are calculated as 0.16, 0.18, 0.53 and 0.13. Finally, the interest rate and tax rate are considered to be in line with the development goal of the supply chain and are consistent with the requirements of social development at 10% and 25%, respectively.

## 4. Discussion

### 4.1. Analysis of Calculation Results

To find the appropriate values of $\varepsilon_{2}$ and $\varepsilon_{3}$, the second and third objective functions are optimized, and their values are denoted as μ and ν, respectively. Then, the values associated with ε are listed. The ranges of μ and ν can be identified, then a Pareto solution might be obtained for each group of μ and ν, and finally, all the results can form a Pareto frontier. 

**Figure 5:**
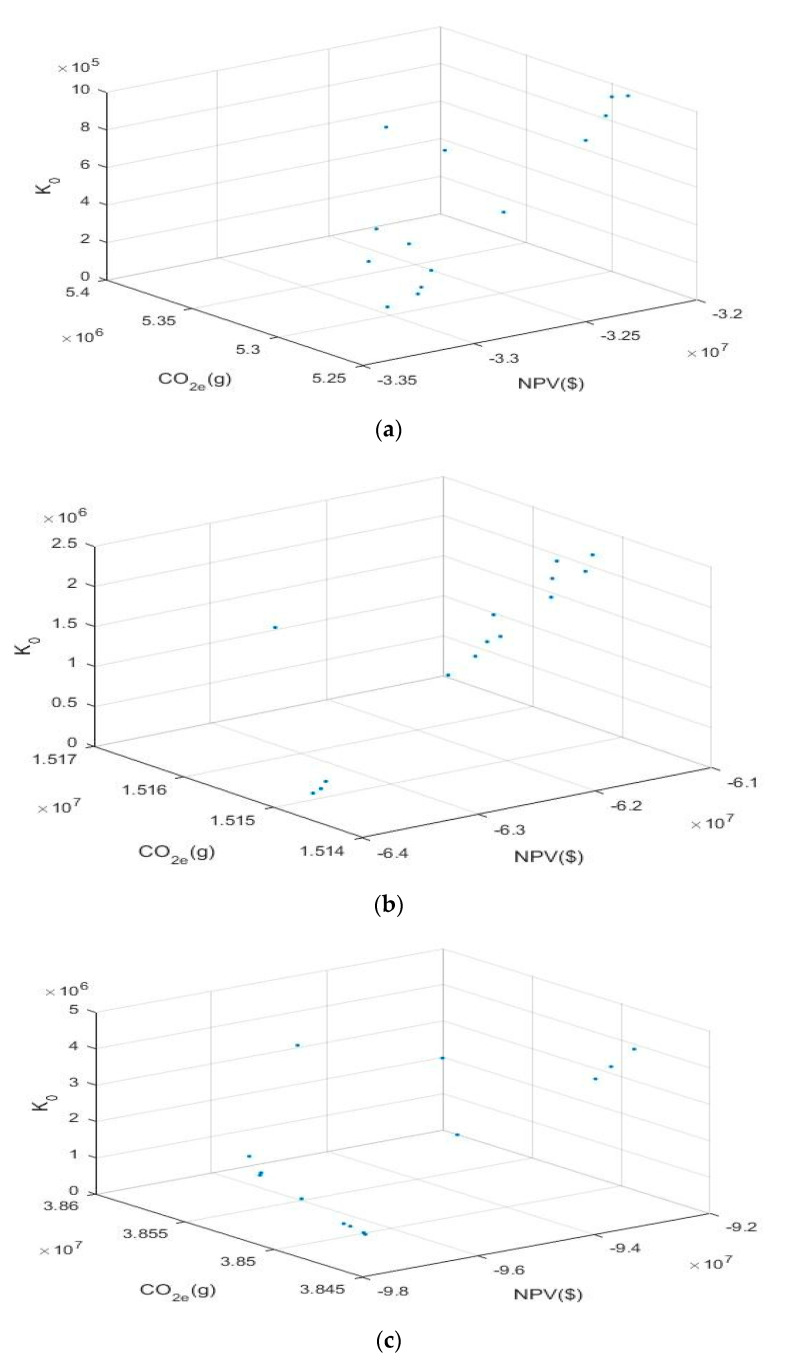
(**a**) The 3D distribution of problem 1. (**b**) The 3D distribution of problem 2. (**c**). The 3D distribution of problem 3.

Next, the model is solved, three sets of Pareto solutions for the test problems are provided in Table 13, Table 14 and Table 15, which illustrates the objective function values for the three differently sized problems (problem 1, 2 and 3). To facilitate the observation of the change in the objective function value sets, three three-dimensional objective function value graphs developed from the three solution sets are shown in Figure 5. The number of variables and the calculation time vary depending on the size of the test problem. Compared to those of the small-sized problem, the variables of the large-sized problem are numerous, and the large problem is relatively difficult to solve.

From Table 13, Table 14 and Table 15, it can be found that the objective functions are contradictory. For the small-sized problem, the ranges of the three objective functions are [−3.31, −3.20] (10^7^), [5.29, 5.36] (10^6^) and [0.91, 9.71] (10^5^). The values of the three objective functions all show oscillating states. In addition, the values of the first two objective functions fluctuate by approximately 3.25 (10^7^) and 5.32 (10^6^); in other words, the fluctuation amplitude is weak, and it is basically stable near a certain fixed value. However, the fluctuation amplitude of the third objective function value is relatively large, and there is no obvious vibration law. Similarly, for the medium-sized problem, the ranges of the three objective functions are [−6.39, −6.14] (10^7^), [1.51, 1.52] (10^7^) and [0.28, 2.43] (10^6^); the fluctuation amplitude of the third objective function value shows a larger increase, and the other objective function values are still relatively stable. Finally, for the large-sized problem, the ranges of the three objective functions are [−9.73, −9.28] (10^7^), [3.85, 3.86] (10^7^) and [0.70, 4.51] (10^6^); the first two objective functions are still stable, the fluctuation amplitude of the third objective function value is also strengthened, and the degree of strength is obvious.

The change in the objective function values of the three differently sized test problems can be observed more intuitively in Figure 5. From Figure 5, it can be seen that, for the three sizes of test problems studied, the distance between the Pareto solutions and the ideal point is different. The distance in the large-sized problem is the largest, the distance of the medium-sized problem is second, and the distance of the small-sized problem is the smallest. Therefore, the proposed ε-constraint method is effective, better results can be obtained when solving small-sized problems.

**Figure 6:**
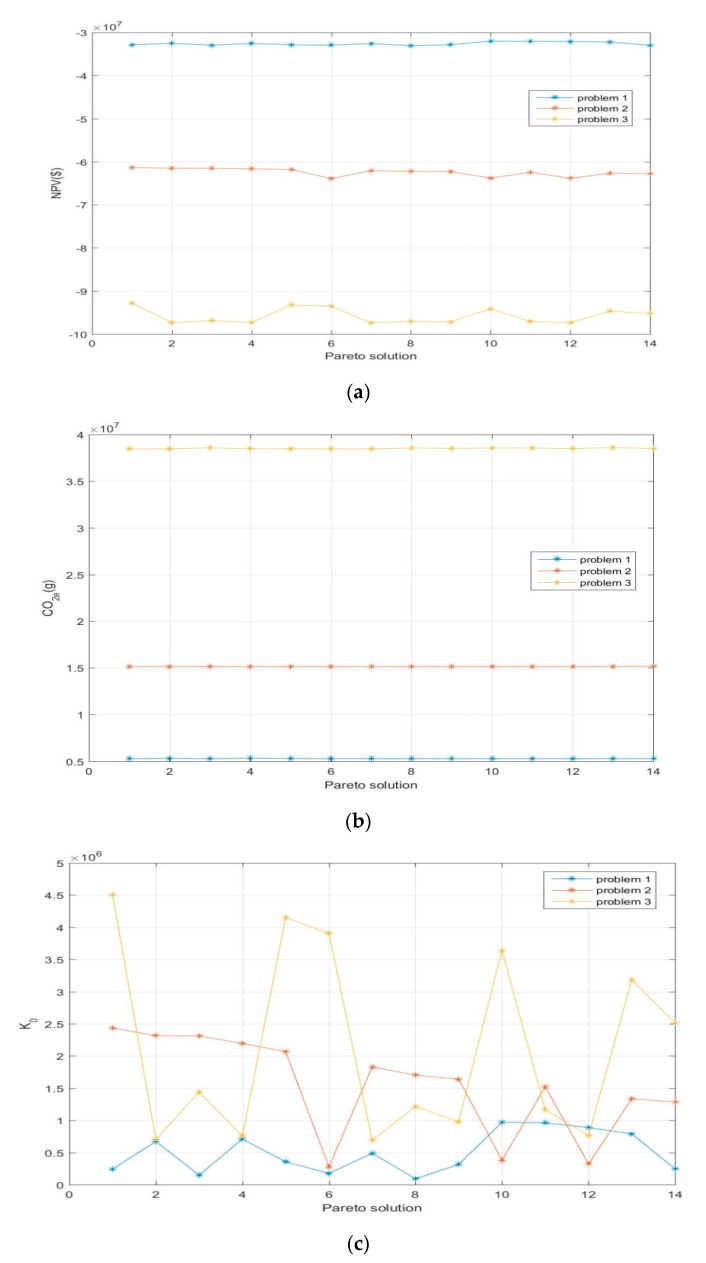
(**a**) Comparison for three sizes of problems on the net present value (NPV). (**b**) Comparison for three sizes of problems on $CO_{2e}$. (**c**) Comparison for three sizes of problems on $K_{0}$.

To compare the variation in the objective function values for all sizes of problems, the results shown in Table 13, Table 14 and Table 15 are converted into the visual map shown in Figure 6. For the first objective function, the decrease in the NPV is due to the increase in the number of entities. The larger the scale of production is, the more customer demand will increase. For the second objective function, by increasing the size of the supply chain, the number of trucks required increases, and the frequency of transportation between entities increases, which will undoubtedly produce more $CO_{2e}$ emissions and waste pollution. For the third objective function, the regional index will change with the change in the size of the problem. The larger the problem size is, the more social impact it has and the more social welfare it creates. In addition, the supply chain network will generate a higher level of social responsibility, and ultimately, the increase in the regional index promotes the increase of the social objective function. 

### 4.2. Sensitivity Analysis

A sensitivity analysis based on two parameters is provided, analysis of the medium-sized test problem is carried out. The range of fluctuations in product demand is [−50%,50%], and the return rate for returned products ranges from 0 to 1.

(1) Based on product demand fluctuation

**Figure 7:**
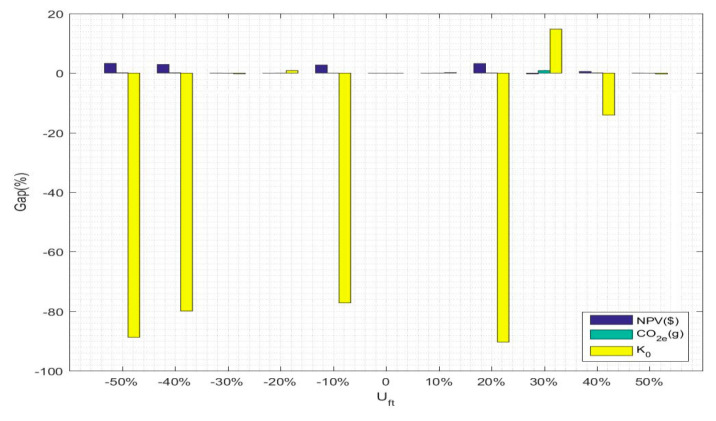
Graphical representation of the % change in objective function value relative to product demand fluctuations.

The results are shown in Table 16. To analyze the influence trend more intuitively, the sensitivity analysis results are converted into a trend graph, as shown in Figure 7. As seen from the table and the figure, the fluctuations in product demand have an impact on the objective functions, with the most serious effect on objective function 3, followed by the objective function 1; the influence on the objective function 2 is almost negligible. For the first objective function, when the product demand is reduced by 20% or increased by 10% and 30% relative to the base product demand, the change in the NPV value is negative; however, for other product demand fluctuations, the change in the NPV value is positive. In addition, when the product demand is reduced by 50% or increased by 20% relative to the base product demand, the changes in the NPV value are the largest at 3.2508% and 3.2017%, respectively. For the second objective function, when the product demand is reduced by 30% or increased by 50% relative to the base product demand, the change in $CO_{2e}$ emissions is negative; i.e., when the product demand is reduced by 30% relative to the base product demand, $CO_{2e}$ emissions are at their lowest level. For the third objective function, the change in the function value is most obvious when the product demand fluctuates, and with respect to the positive change in social benefits, when the product demand is increased by 30% over the base product demand, the social benefits value changes the most, with an additional social benefit value of 14.7580% over that of the original product demand.

(2) Based on the fluctuation of the return rate for returned products

**Figure 8:**
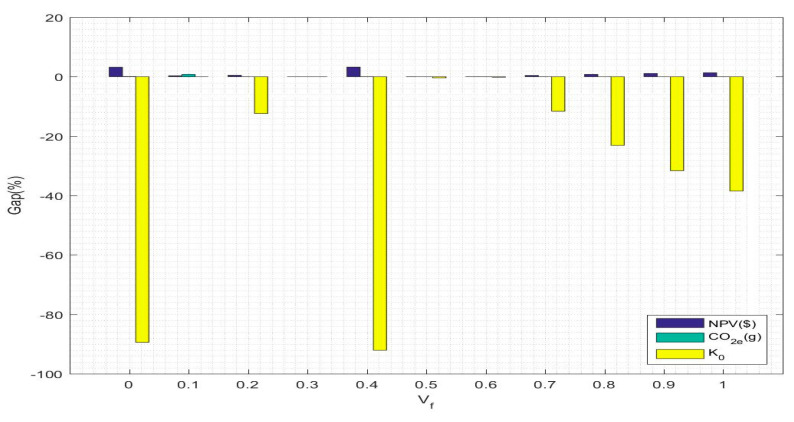
Graphical representation of the % change in objective function value relative to return rate fluctuations.

The results are shown in Table 17, and a graphical representation is shown in Figure 8. As seen from the table and the figure, fluctuations in the return rate have an impact on the objective functions, and the phenomenon is consistent with the ranking of influence on the objective function due to product demand fluctuations. For the first objective function, the base return rate is 0.3; however, when the existing return rate is changed to 0.4, the change in the NPV value is the largest and reaches 3.2487%. For the second objective function, when the return rate changes, it has less influence on $CO_{2e}$ emissions, and the objective function value basically fluctuates within a small range of approximately 1.52 (10^7^). When the existing return rate is changed to one, the negative change in $CO_{2e}$ emissions reaches the maximum range; that is, there is the smallest amount of $CO_{2e}$ emissions when the used products are all collected. For the third objective function, when the return rate is 0.1, the change in social benefits is positive, while the changes in the social benefits of the other return rates are negative. In addition, when the return rate is 0.1, the social benefit value reaches its peak.

### 4.3. Challenges and Limitations

The model is considered novel and effective compared with the previous research results. The novelty is mainly reflected in two aspects. Firstly, this paper combines the proposed global closed-loop supply chain model with dual channel sales and dual channel collection model, which is more in line with the status of online and offline linkage operation for the supply chain network, under the background of big data driven. At present, scholars’ research on the global closed-loop supply chain only stays at the level of qualitative analysis. Compared with the traditional closed-loop supply chain, the more complex and intelligent internal operation process has not been carefully studied and discussed. Secondly, based on the previous research on the recycling of waste products, this paper focuses on the impact of the quality level of returned products on the recycling. The quality of recycled products is divided into three different levels, and the remanufacturing process is optimized based on the fact that recycled products of different quality can produce different quantities of parts, which makes up for the existing research blank. The effectiveness is mainly reflected in the good effect of the proposed ε-constraint method to solve the model. From Figure 5, it finds that the fitting effect of the proposed model is different for different size problems. In addition, Figure 6 shows that the stability of the objective function value obtained by solving the model is different when the problem size is different. Generally speaking, the proposed model can achieve ideal results for solving small-scale closed-loop supply chain problems in this paper. Finally, the change of product demand and waste recovery rate will also have a different impact on the objective function. The most desired result can be obtained according to the changing trend of the two.

However, there are still some limitations to be further studied by other scholars. First, when an existing company experiences change in the operating structure, the network structure of the dual-channel closed supply chain should be adjusted and developed to adapt to the future development direction of the company. Second, the design problem of a single-product model is studied in this paper; however, companies often produce and sell all types of products, and a product is usually updated with the development and advancement of technology. The single-product model no longer conforms to the production model of existing companies, so a multi-product optimization model needs to be considered in further research. Additionally, the uncertainty of the parameters is not considered. The dynamics and complexity of the supply chain greatly increase the uncertainty of decisions, which significantly affects the performance of the entire supply chain network. Therefore, stochastic programming can be used to deal with the uncertainty of the parameters in future research.

## 5. Conclusions

This paper aims to maximize the net present value of the system, minimize $CO_{2e}$ emissions from supply chain activities, and maximize the closed-loop manufacturing social sustainability index. This research proposes a single-product, multi-period, multi-objective mixed integer linear programming model to design and plan the global closed-loop supply chain network considering dual-channel sales and dual-channel collection, aiming to fill the gaps in existing research. Dual-channel sales and collection respectively refer to the combination of online and offline sales and collection channels. This is very common in life, but it is usually not fully considered in academic research due to complexity. Therefore, the research results of this paper have important reference value and significance for the design and optimization of real-life closed-loop supply chain networks. The conclusions are as follows.

(1) The proposed ε-constraint method can converge with a probability of one to the global optimal solution set.
(2) The proposed ε-constraint method can be used to solve optimization problems of different sizes, but better results can be obtained when solving small-sized problems.
(3) The first objective function value is inversely proportional to the problem size. The second is proportional to the problem size. The third is not proportional to the problem size; however, in general, the larger the problem size, the higher the extreme value.
(4) Fluctuations in product demand have an impact on the objective functions, with the most serious effect on objective function 3, followed by objective function 1; the influence on objective function 2 is almost negligible.
(5) The fluctuations in the return rate also have an impact on the objective functions, and the phenomenon is consistent with the ranking of influence on the objective function due to product demand fluctuations.

## Figures and Tables

**Table 1:** The sets used.

Sets	Definition
*a*	Set of components, a=1,2,…,A
*b*	Set of CPUs, b=1,2,…,B
*c*	Set of factories, c=1,2,…,C
*d*	Set of warehouses, d=1,2,…,D
*e*	Set of retailers, e=1,2,…,E
*f*	Set of consumers, f=1,2,…,F
*g*	Set of collection centers, g=1,2,…,G
*h*	Set of disassembly centers, h=1,2,…,H
*i*	Set of refurbishing centers, i=1,2,…,I
*j*	Set of production machines, j=1,2,…,J
*k*	Set of returned product’s quality, k=1,2,…,K
*m*	Set of entities within the analysis boundary (Retailers are not included), m=1,2,…,M
*t*	Set of time periods, t=1,2,…,T

**Table 2:** Product and component flow-related parameters.

Decision Variables	Definition
$A_{b}^{0}$	$\{\begin{array}{l} 1,\mathrm{if}\;\mathrm{component}\;\mathrm{production}\;\mathrm{unit}\;b\;\mathrm{is}\;\mathrm{established}\\ \;0,\mathrm{otherwise} \end{array}$
$A_{c}^{0}$	$\{\begin{array}{l} 1,\mathrm{if}\;\mathrm{factory}\;c\;\mathrm{is}\;\mathrm{established}\\ 0,\mathrm{otherwise} \end{array}$
$A_{d}^{0}$	$\{\begin{array}{l} 1,\mathrm{if}\;\mathrm{warehouse}\;d\;\mathrm{is}\;\mathrm{established}\\ 0,\mathrm{otherwise} \end{array}$
$A_{e}^{0}$	$\{\begin{array}{l} 1,\mathrm{if}\;\mathrm{retailer}\;e\;\mathrm{is}\;\mathrm{rented}\\ 0,\mathrm{otherwise} \end{array}$
$A_{g}^{0}$	$\{\begin{array}{l} 1,\mathrm{if}\;\mathrm{collection}\;\mathrm{center}\;g\;\mathrm{is}\;\mathrm{established}\\ 0,\mathrm{otherwise} \end{array}$
$A_{h}^{0}$	$\{\begin{array}{l} 1,\mathrm{if}\;\mathrm{disassembly}\;\mathrm{center}\;h\;\mathrm{is}\;\mathrm{established}\\ 0,\mathrm{otherwise} \end{array}$
$A_{i}^{0}$	$\{\begin{array}{l} 1,\mathrm{if}\;\mathrm{refurbishing}\;\mathrm{center}\;i\;\mathrm{is}\;\mathrm{established}\\ 0,\mathrm{otherwise} \end{array}$
$B_{abct}^{0}$	Number of component *a* shipped from CPU *b* to factory *c* in time period *t*
$B_{cdt}^{0}$	Number of products shipped from factory *c* to warehouse *d* in time period *t*
$C_{det}^{0}$	Number of products sold from warehouse *d* to retailer *e* in time period *t*
$C_{dft}^{0}$	Number of products sold from warehouse *d* to consumer *f* in time period *t*
$C_{eft}^{0}$	Number of products sold from retailer *e* to consumer *f* in time period *t*
$D_{fet}^{0}$	Number of returned products collected from consumer *f* to retailer *e* in time period *t*
$D_{kfet}^{0}$	Number of returned products with quality *k* for consumer *f* to retailer *e* in time period *t*
$D_{fgt}^{0}$	Number of returned products collected from consumer *f* to collection center *g* in time period *t*
$D_{kfgt}^{0}$	Number of returned products with quality *k* for consumer *f* to collection center *g* in time period *t*
$D_{egt}^{o}$	Number of returned products collected from retailer *e* to collection center *g* in time period *t*
$D_{kegt}^{0}$	Number of returned products with quality *k* for retailer *e* to collection center *g* in time period *t*
$E_{gct}^{0}$	Number of returned products shipped from collection center *g* to factory *c* in time period *t*
$E_{ght}^{0}$	Number of returned products shipped from collection center *g* to disassembly center *h* in time period *t*
$F_{ahit}^{0}$	Number of component *a* shipped from disassembly center *h* to refurbishing center *i* in time period *t*
$F_{aht}^{0}$	Number of component *a* shipped from disassembly center *h* to recycle center in time period *t*
$G_{aht}^{0}$	Number of component *a* shipped from disassembly center *h* to disposal center in time period *t*
$G_{aict}^{0}$	Number of component *a* shipped from refurbishing center *i* to factory *c* in time period *t*
$H_{dt}^{0}$	Inventory of products in warehouse *d* at the end of time period *t*
$H_{d\left(t-1\right)}^{0}$	Inventory of products in warehouse *d* at the end of time period *t*−1
$I_{aht}^{0}$	Number of component *a* obtained at disassembly center *h* in time period *t*
$J_{ct}^{0}$	Number of returned products for minor repairs at factory *c* in time period *t*
$J_{jct}^{0}$	Number of returned products for minor repairs with production machine *j* at factory *c* in time period *t*
$K_{ct}^{0}$	Number of products produced at factory *c* in time period *t*
$K_{jct}^{0}$	Number of products produced with production machine *j* at factory *c* in time period *t*
$L_{ct}^{0}$	Number of products remanufactured at factory *c* in time period *t*
$L_{jct}^{0}$	Number of products remanufactured with production machine *j* at factory *c* in time period *t*

**Table 3:** The social sustainability criteria for the dual-channel global closed-loop supply chains.

Criteria	References	Description
Extended producer responsibilities ($C_{1}$)	Gui et al. [47]	This mainly refers to a series of measures taken by producers in the process of promoting product collection, including stimulating consumers to return returned products, collection from discarded products, etc.
Employment practice ($C_{2}$)	Furlan et al. [48]	This means that employment skills can be improved through employee training and exercise, and then more employment opportunities are provided to local residents.
Economic welfare and growth ($C_{3}$)	Zhu et al. [49]	This refers to the economic impact of the system on the region where the system is located, mainly including the impact on local education concepts, employment concepts, and the impact on the lives of surrounding residents.
Responsibilities towards stakeholders ($C_{4}$)	Mathivathanan et al. [50]	This refers to the tasks performed by internal members of the system to maintain system operation and stability, such as regular reporting of progress and plans, supervision of processing and manufacturing activities, as well as coordination between various departments and members, etc.

**Table 4:** Pairwise comparison matrix between social sustainability criteria and the goal [46,51].

Goal	$C_{1}$	$C_{2}$	$C_{3}$	$C_{4}$
$C_{1}$	1	3	1/5	1/2
$C_{2}$	1/3	1	1/2	3
$C_{3}$	5	2	1	4
$C_{4}$	2	1/3	1/4	1

**Table 5:** The sizes of the test problems.

Sets	Small Size	Medium Size	Large Size
B	C1	C1, C2	C1, C2, C3
C	F1	F1, F2	F1, F2, F3, F4
D	W1	W1	W1, W2
E	R1	R1, R2	R1, R2, R3, R4
F	O1 O2	O1, O2, O3	O1, O2, O3, O4
G	L1	L1, L2	L1, L2, L3
H	D1	D1, D2	D1, D2
I	E1	E1, E2, E3	E1, E2, E3, E4

**Table 6:** The components and production machine list for products.

	FP	RP1	RP2	RP3
M1	5	5	2	1
M2	4	4	2	1
M3	7	7	3	2
M4	6	6	3	1
Production machines	P1, P2, P3, P4	-	P1, P3	P2, P3

**Table 7:** Entity capacity characteristics.

	CPU		Factory		Warehouse	Retailer		Collection Center	Disassembly Center		Refurbishing Center
	$A_{ab}^{\max}$	$A_{ab}^{\min}$	$A_{ac}^{\max}$	$A_{c}^{\max}$	$A_{d}^{\max}$	$A_{e1}^{\max}$	$A_{e2}^{\max}$	$A_{g}^{\max}$	$A_{h}^{\max}$	$A_{ah}^{\max}$	$A_{ai}^{\max}$
M1	3600	3500	3500	-	-	-	-	-	-	3000	4200
M2	2000	1500	2000	-	-	-	-	-	-	3000	3600
M3	4500	4000	4400	-	-	-	-	-	-	3500	4700
M4	4000	3500	4200	-	-	-	-	-	-	3200	5000
FP	-	-	-	1000	1500	800	-	-	-	-	-
RP	-	-	-	-	-	-	200	1500	2000	-	-

**Table 8:** Cost, revenue and proportion characteristics associated with components ($).

	Production Cost	Refurbishment Cost	Recycle Revenue	Disposal Cost	Transportation Cost					Proportion (%)		
	$B_{ab}$	$B_{a1}$	$B_{a2}$	$B_{a3}$	$D_{abc}$	$D_{ahi}$	$D_{ah1}$	$D_{ah2}$	$D_{aic}$	$N_{a1}^{\max}$	$N_{a2}^{\max}$	$N_{a3}^{\max}$
M1	7	2	0.5	0.1	0.5	0.3	0.4	0.5	0.3	30	35	40
M2	8	2	0.5	0.3	0.2	0.3	0.6	0.5	0.2	35	40	25
M3	6	3	0.5	0.2	0.3	0.2	0.5	0.4	0.2	20	30	60
M4	6	4	0.5	0.2	0.4	0.4	0.3	0.5	0.4	25	40	40

**Table 9:** Cost characteristics associated with final products and returned products.

	Disassembly Cost ($)	Collection Cost ($)		Operating Cost	Transportation Cost						
	$B_{0}$	$C_{kfg}$	$C_{kfe}$	$C_{j}$	$D_{cd}$	$D_{de}$	$D_{df}$	$D_{fg}$	$D_{eg}$	$D_{gc}$	$D_{gh}$
FP	-	-	-	16,13,12,18	0.005	0.005	0.003	-	-	-	-
RP1	-	80	80	-	-	-	-	0.003	0.002	0.004	-
RP2	8	50	50	16,13,12,18	-	-	-	0.003	0.002	-	0.003
RP3	8	30	30	16,13,12,18	-	-	-	0.003	0.002	-	0.003

**Table 10:** Entity construction and worker characteristics.

	Construction		Worker	
	Cost ($)	$CO_{2e}\mathrm{Emissions}\;\mathrm{Amount}\;(g)$	Wage in a Week ($)	Number
CPU	10,000	1200	1400	40
Factory	12,000	1400	1600	45
Warehouse	11,000	500	1200	16
Retailer	-	-	1000	22
Collection center	6000	600	1100	36
Disassembly center	8000	800	1200	35
Refurbishing center	9000	900	1200	35

**Table 11:** Distance characteristics between entities (km).

	CPU	Warehouse	Collection Center	Refurbishing Center	Recycle Center	Disposal Center
Factory	800	300	900	200	-	-
Retailer	-	1000	100	-	-	-
Consumer	-	500	200	-	-	-
Disassembly center	-	-	500	100	600	700

**Table 12:** $CO_{2e}$ emissions amount associated with supply chain activities.

	Production	Refurbishing	Disposal		Production	Remanufacturing	Minor Repair	Disassembling
	$L_{a}$	$L_{a1}$	$L_{a2}$		$L_{1}$	$L_{2}$	$L_{3}$	$L_{4}$
M1	20	3	5	FP	80	-	-	-
M2	30	2	6	RP1	-	-	40	-
M3	15	4	8	RP2	-	70	-	60
M4	25	3	5	RP3	-	70	-	60

**Table 13:** The results of problem 1.

Pareto Solution	$\varepsilon_{2}$	$\varepsilon_{3}$	$\varepsilon_{2}$ (10^6^)	$\varepsilon_{3}$ (10^6^)	NPV ($) (10^7^)	$CO_{2e}(g)$ (10^6^)	$K_{0}$ (10^5^)
1	μ+1∗μ	ν+1∗ν	10.70	1.57	−3.29	5.29	2.41
2	μ+0.8∗μ	ν+0.9∗ν	9.65	1.49	−3.25	5.33	6.72
3	μ+0.75∗μ	ν+0.9∗ν	9.38	1.49	−3.30	5.29	1.50
4	μ+0.7∗μ	ν+0.8∗ν	9.11	1.41	−3.25	5.36	7.09
5	μ+0.65∗μ	ν+0.7∗ν	8.85	1.34	−3.29	5.32	3.56
6	μ+0.6∗μ	ν+0.6∗ν	8.58	1.26	−3.30	5.29	1.76
7	μ+0.55∗μ	ν+0.5∗ν	8.31	1.18	−3.26	5.29	4.89
8	μ+0.5∗μ	ν+0.4∗ν	8.04	1.10	−3.31	5.29	0.91
9	μ+0.45∗μ	ν+0.35∗ν	7.77	1.06	−3.28	5.31	3.16
10	μ+0.45∗μ	ν+0.3∗ν	7.77	1.02	−3.20	5.29	9.71
11	μ+0.4∗μ	ν+0.25∗ν	7.51	0.98	−3.20	5.29	9.61
12	μ+0.4∗μ	ν+0.2∗ν	7.51	0.94	−3.21	5.29	8.88
13	μ+0.2∗μ	ν+0.1∗ν	6.43	0.86	−3.22	5.29	7.90
14	μ+0∗μ	ν+0∗ν	5.36	0.79	−3.30	5.31	2.46

**Table 14:** The results of problem 2.

Pareto Solution	$\varepsilon_{2}$ (10^7^)	$\varepsilon_{3}$ (10^6^)	NPV ($) (10^7^)	$CO_{2e}(g)$ (10^7^)	$K_{0}$ (10^6^)
1	3.05	2.44	−6.14	1.51	2.43
2	2.75	2.31	−6.15	1.52	2.32
3	2.67	2.31	−6.15	1.51	2.31
4	2.59	2.19	−6.16	1.51	2.19
5	2.52	2.07	−6.18	1.51	2.07
6	2.44	1.95	−6.39	1.51	0.28
7	2.36	1.83	−6.21	1.52	1.83
8	2.29	1.71	−6.22	1.51	1.7
9	2.21	1.64	−6.23	1.51	1.64
10	2.21	1.58	−6.38	1.51	0.38
11	2.14	1.52	−6.24	1.51	1.52
12	2.14	1.46	−6.39	1.51	0.32
13	1.83	1.34	−6.27	1.51	1.34
14	1.53	1.22	−6.28	1.52	1.28

**Table 15:** The results of problem 3.

Pareto Solution	$\varepsilon_{2}$ (10^7^)	$\varepsilon_{3}$ (10^6^)	NPV ($) (10^7^)	$CO_{2e}(g)$ (10^7^)	$K_{0}$ (10^6^)
1	7.73	4.88	−9.28	3.85	4.51
2	6.96	4.64	−9.73	3.85	0.70
3	6.77	4.64	−9.68	3.86	1.44
4	6.57	4.39	−9.73	3.85	0.76
5	6.38	4.15	−9.32	3.85	4.15
6	6.19	3.91	−9.35	3.85	3.91
7	5.99	3.66	−9.73	3.85	0.69
8	5.80	3.42	−9.70	3.85	1.21
9	5.61	3.30	−9.72	3.85	0.98
10	5.61	3.17	−9.41	3.85	3.64
11	5.41	3.05	−9.70	3.85	1.17
12	5.41	2.93	−9.73	3.85	0.76
13	4.64	2.69	−9.47	3.86	3.18
14	3.87	2.44	−9.52	3.85	2.52

**Table 16:** Sensitivity analysis results for product demand fluctuations.

	NPV ($)		$CO_{2e}(g)$		$K_{0}$	
$U_{ft}$	Value (10^7^)	Gap (%)	Value (10^7^)	Gap (%)	Value (10^6^)	Gap (%)
−50%	−6.41	3.2508	1.52	0.0984	0.21	−88.7650
−40%	−6.39	2.9003	1.52	0.0853	0.37	−79.9650
−30%	−6.21	00.0026	1.51	−0.0229	1.83	−0.2443
−20%	−6.21	−0.0003	1.52	0.0029	1.85	0.8410
−10%	−6.38	2.7087	1.52	0.0045	0.42	−77.2150
0 (Base demand)	−6.21	0	1.52	0	1.83	0
10%	−6.21	−0.0031	1.52	0.0063	1.83	0.1543
20%	−6.41	3.2017	1.52	0.0459	0.18	−90.3700
30%	−6.19	−0.2764	1.53	0.8188	2.10	14.7580
40%	−6.24	0.5472	1.52	0.0779	1.57	−14.1280
50%	−6.21	0.0049	1.51	−0.0159	1.82	−0.3211

Notes: “Value” refers to the objective function value, and “Gap” refers to the percentage change for the objective function value of the changed product demand relative to the base product demand.

**Table 17:** Sensitivity analysis results for return rate fluctuations.

$V_{f}\;$	NPV ($)		$CO_{2e}(g)$		$K_{0}\;$	
	Value (10^7^)	Gap (%)	Value (10^7^)	Gap (%)	Value (10^6^)	Gap (%)
0	−6.41	3.1925	1.52	0.0634	0.19	−89.4250
0.1	−6.23	0.3133	1.53	0.7619	1.83	0.0002
0.2	−6.24	0.4761	1.52	0.0114	1.60	−12.4020
0.3 (Base rate)	−6.21	0	1.52	0	1.83	0
0.4	−6.41	3.2487	1.52	0.0329	0.15	−92.0880
0.5	−6.21	0.0094	1.51	−0.0133	1.82	−0.4200
0.6	−6.21	0.0067	1.51	−0.0190	1.83	−0.1923
0.7	−6.23	0.4101	1.52	0.0098	1.62	−11.6010
0.8	−6.26	0.7972	1.51	−0.0163	1.41	−23.1130
0.9	−6.28	1.0942	1.51	−0.0145	1.25	−31.6300
1	−6.29	1.3339	1.51	−0.0221	1.13	−38.4850

Notes: “Value” refers to the objective function value, and “Gap” refers to the percentage change for the objective function value of the changed return rate relative to the base return rate.

## Appendix A

**Table A1:** Supply and capacity parameters.

Parameters	Definition
$A_{ab}^{\max}$	Maximum supply of component *a* by CPU *b*
$A_{ab}^{\min}$	Minimum supply of component *a* by CPU *b*
$A_{ac}^{\max}$	Maximum capacity for component *a* in factory *c*
$A_{c}^{\max}$	Maximum capacity for products in factory *c*
$A_{d}^{\max}$	Maximum inventory capacity for products in warehouse *d*
$A_{e1}^{\max}$	Maximum capacity for products in retailer *e*
$A_{e2}^{\max}$	Maximum capacity for returned products in retailer *e*
$A_{g}^{\max}$	Maximum capacity for returned products in collection center *g*
$A_{h}^{\max}$	Maximum capacity for returned products in disassembly center *h*
$A_{ah}^{\max}$	Maximum capacity for component *a* in disassembly center *h*
$A_{ai}^{\max}$	Maximum capacity for component *a* in refurbishing center *i*

**Table A2:** Cost, revenue and price parameters.

Parameters	Definition
$B_{ab}$	Production cost per unit of component *a* at CPU *b*
$B_{dt}$	Inventory holding cost per unit of product at warehouse *d* in time period *t*
$B_{0}$	Unit disassembly cost of returned products
$B_{a1}$	Unit refurbishment cost of component *a*
$B_{a2}$	Unit recycle revenue of component *a*
$B_{a3}$	Unit disposal cost of component *a*
$C_{kfg}$	Unit collection cost of returned products with quality *k* from consumer *f* to collection center *g*
$C_{kfe}$	Unit collection cost of returned products with quality *k* from consumer *f* to retailer *e*
$C_{1t}$	Sale price per unit of product from retailers to consumers in time period *t*
$C_{2t}$	Sale price per unit of product from warehouses to consumers in time period *t*
$C_{j}$	Operating cost per unit of product on production machine *j*
$D_{abc}$	Transportation cost per kilometer per unit of component *a* from CPU *b* to factory *c*
$D_{cd}$	Transportation cost per kilometer per unit of product from factory *c* to warehouse *d*
$D_{de}$	Transportation cost per kilometer per unit of product from warehouse *d* to retailer *e*
$D_{df}$	Transportation cost per kilometer per unit of product from warehouse *d* to consumer *f*
$D_{fg}$	Transportation cost per kilometer per unit of returned product from consumer *f* to collection center *g*
$D_{eg}$	Transportation cost per kilometer per unit of returned product from retailer *e* to collection center *g*
$D_{gc}$	Transportation cost per kilometer per unit of returned product from collection center *g* to factory *c*
$D_{gh}$	Transportation cost per kilometer per unit of returned product from collection center *g* to disassembly center *h*
$D_{ahi}$	Transportation cost per kilometer per unit of component *a* from disassembly center *h* to refurbishing center *i*
$D_{ah1}$	Transportation cost per kilometer per unit of component *a* from disassembly center *h* to recycle center
$D_{ah2}$	Transportation cost per kilometer per unit of component *a* from disassembly center *h* to disposal center
$D_{aic}$	Transportation cost per kilometer per unit of component *a* from refurbishing center *i* to factory *c*
$E_{b}$	Fixed construction cost of CPU *b*
$E_{c}$	Fixed construction cost of factory *c*
$E_{d}$	Fixed construction cost of warehouse *d*
$E_{g}$	Fixed construction cost of collection center *g*
$E_{h}$	Fixed construction cost of disassembly center *h*
$E_{i}$	Fixed construction cost of refurbishing center *i*
$F_{et}$	Fixed rental cost for retailer *e* in the time period *t*
$F_{m}$	Fixed original investment of entity *m*
$F_{j}$	Fixed original investment of production machine *j*
$F_{0}$	Fixed original investment of truck transport
$G_{t}$	Net cash flow during time period *t*
$H_{t}$	Net profit in time period *t*
$I_{t}$	Total revenue in time period *t*
$J_{t}$	Total cost in time period *t*
$K_{0}$	Social sustainability indicator

**Table A3:** $CO_{2e}$ emissions amount related parameters.

Parameters	Definition
$L_{b}$	$CO_{2e}$ emissions amount for the construction of CPU *b*
$L_{c}$	$CO_{2e}$ emissions amount for the construction of factory *c*
$L_{d}$	$CO_{2e}$ emissions amount for the construction of warehouse *d*
$L_{g}$	$CO_{2e}$ emissions amount for the construction of collection center *g*
$L_{h}$	$CO_{2e}$ emissions amount for the construction of disassembly center *h*
$L_{i}$	$CO_{2e}$ emissions amount for the construction of refurbishing center *i*
$L_{a}$	$CO_{2e}$ emissions amount for producing per unit of component *a* at CPUs
$L_{1}$	$CO_{2e}$ emissions amount for producing per unit of product at factories
$L_{2}$	$CO_{2e}$ emissions amount for remanufacturing per unit of product at factories
$L_{3}$	$CO_{2e}$ emissions amount for minor repairs of per unit of returned product at factories
$L_{4}$	$CO_{2e}$ emissions amount for disassembling per unit returned product at disassembly centers
$L_{a1}$	$CO_{2e}$ emissions amount for refurbishing per unit component *a* at refurbishing centers
$L_{a2}$	$CO_{2e}$ emissions amount for disposing pert unit component *a* at disposal center
$L_{0}$	$CO_{2e}$ emissions amount per kilometer of truck transport

**Table A4:** Distance related parameters.

Parameters	Definition
$M_{bc}$	Distance between CPU *b* and factory *c*
$M_{cd}$	Distance between factory *c* and warehouse *d*
$M_{de}$	Distance between warehouse *d* and retailer *e*
$M_{df}$	Distance between warehouse *d* and consumer *f*
$M_{fg}$	Distance between consumer *f* and collection center *g*
$M_{eg}$	Distance between retailer *e* and collection center *g*
$M_{gc}$	Distance between collection center *g* and factory *c*
$M_{gh}$	Distance between collection center *g* and disassembly center *h*
$M_{hi}$	Distance between disassembly center *h* and refurbishing center *i*
$M_{h1}$	Distance between disassembly center *h* and recycle center
$M_{h2}$	Distance between disassembly center *h* and disposal center
$M_{ic}$	Distance between refurbishing center *i* and factory *c*

**Table A5:** Other parameters.

Parameters	Definition
$K_{1}$	Regional index of extended producer responsibilities
$K_{2}$	Regional index of employment practice
$K_{3}$	Regional index of economic welfare and growth
$K_{4}$	Regional index of responsibilities towards stakeholders
$w_{1}$	Relative weight of extended producer responsibilities
$w_{2}$	Relative weight of employment practice
$w_{3}$	Relative weight of economic welfare and growth
$w_{3}$	Relative weight of responsibilities towards stakeholders
$N_{a1}^{\max}$	Maximum proportion of component *a* refurbishment
$N_{a2}^{\max}$	Maximum proportion of component *a* recycling
$N_{a3}^{\max}$	Maximum proportion of component *a* disposal
$O_{j}$	Number of workers required for production machine *j*
$P_{1}$	Fixed wage of a worker at CPUs in a week
$P_{2}$	Fixed wage of a worker at factories in a week
$P_{3}$	Fixed wage of a worker at warehouses in a week
$P_{4}$	Fixed wage of a worker at retailers in a week
$P_{5}$	Fixed wage of a worker at collection centers in a week
$P_{6}$	Fixed wage of a worker at disassembly centers in a week
$P_{7}$	Fixed wage of a worker at refurbishing centers in a week
$Q_{0}$	Number of time periods in the time range
$Q_{t}$	Number of weeks in time period *t*
$Q_{b}$	Number of workers required for CPU *b*
$Q_{c}$	Number of workers required for factory *c* (Workers required for production machines are not included)
$Q_{d}$	Number of workers required for warehouse *d*
$Q_{e}$	Number of workers required for retailer *e*
$Q_{g}$	Number of workers required for collection center *g*
$Q_{h}$	Number of workers required for disassembly center *h*
$Q_{i}$	Number of workers required for refurbishing center *i*
$R_{0}$	Interest rate
$S_{0}$	Tax rate
$T_{m}$	Residual value ratio of entity *m*
$T_{j}$	Residual value ratio of production machine *j*
$T_{0}$	Residual value ratio of truck transport
$U_{ft}$	Demand for products by customer *f* in time period *t*
$V_{f}$	Return rate of products at consumer *f*

## Appendix B

Demand constraints

(A1) $$\sum_{d}C_{dft}^{0}+\sum_{e}C_{eft}^{0}=U_{ft}$$

(A2) $$\sum_{g}D_{fgt}^{0}+\sum_{e}D_{fet}^{0}=V_{f}U_{ft}$$

Constraints (A1) and (A2) ensure demand balance for the forward and reverse supply chains.

Flow balance constraints

(A3) $$K_{ct}^{0}+L_{ct}^{0}+J_{ct}^{0}=\sum_{d}B_{cdt}^{0}$$

(A4) $$\sum_{d}C_{det}^{0}=\sum_{f}C_{eft}^{0}$$

(A5) $$\sum_{f}D_{fet}^{0}=\sum_{g}D_{egt}^{0}$$

(A6) $$\sum_{f}D_{fgt}^{0}+\sum_{e}D_{egt}^{0}=\sum_{c}E_{gct}^{0}+\sum_{h}E_{ght}^{0}$$

(A7) $$I_{aht}^{0}=\sum_{i}F_{ahit}^{0}+F_{aht}^{0}+G_{aht}^{0}$$

(A8) $$\sum_{h}F_{ahit}^{0}=\sum_{c}G_{aict}^{0}$$

Constraints (A3)–(A8) ensure flow balance for the components and products of the entities within the analysis boundary.

Entity balance constraints

(A9) $$\sum_{k}D_{kfgt}^{0}=D_{fgt}^{0}$$

(A10) $$\sum_{k}D_{kegt}^{0}=D_{egt}^{0}$$

(A11) $$\sum_{k}D_{kfet}^{0}=D_{fet}^{0}$$

Constraints (A9)–(A11) ensure the balance for returned products of retailers and collection centers.

Production machine balance constraints

(A12) $$\sum_{j}J_{jct}^{0}=J_{ct}^{0}$$

(A13) $$\sum_{j}K_{jct}^{0}=K_{ct}^{0}$$

(A14) $$\sum_{j}L_{jct}^{0}=L_{ct}^{0}$$

Constraints (A12)–(A14) ensure the balance for products of production machine operations, which determine the number of minorly repaired, produced and remanufactured products using different production machines, respectively.

Supply capacity constraints

(A15) $$A_{ab}^{\min}A_{b}^{0}\leq \sum_{c}\sum_{t}B_{abct}^{0}\leq A_{ab}^{\max}A_{b}^{0}$$

Constraint (A15) indicates that the number of components supplied by the CPUs to the factories is limited by the maximum supply and the minimum supply.

Entity capacity constraints

(A16) $$\sum_{b}\sum_{t}B_{abct}^{0}+\sum_{i}\sum_{t}G_{aict}^{0}\leq A_{ac}^{\max}A_{c}^{0}$$

(A17) $$\sum_{t}K_{ct}^{0}+\sum_{t}L_{ct}^{0}+\sum_{t}J_{ct}^{0}\leq A_{c}^{\max}A_{c}^{0}$$

(A18) $$H_{dt}^{0}=H_{d\left(t-1\right)}^{0}+\sum_{c}B_{cdt}^{0}-\sum_{f}C_{dft}^{0}-\sum_{e}C_{det}^{0}$$

(A19) $$\sum_{t}H_{dt}^{0}\leq A_{d}^{\max}A_{d}^{0}$$

(A20) $$\sum_{d}\sum_{t}C_{det}^{0}\leq A_{e1}^{\max}A_{e}^{0}$$

(A21) $$\sum_{f}\sum_{t}D_{fet}^{0}\leq A_{e2}^{\max}A_{e}^{0}$$

(A22) $$\sum_{f}\sum_{t}D_{fgt}^{0}+\sum_{e}\sum_{t}D_{egt}^{0}\leq A_{g}^{\max}A_{g}^{0}$$

(A23) $$\sum_{g}\sum_{t}E_{ght}^{0}\leq A_{h}^{\max}A_{h}^{0}$$

(A24) $$\sum_{t}I_{aht}^{0}\leq A_{ah}^{\max}A_{h}^{0}$$

(A25) $$\sum_{h}\sum_{t}F_{ahit}^{0}\leq A_{ai}^{\max}A_{i}^{0}$$

Constraints (A16)–(A25) ensure the capacity constraints of components, products and returned products within the entities, namely, that the number of components, products and returned products arriving at an entity will remain within the maximum capacity of the entity.

Flow capacity constraints

(A26) $$\sum_{i}F_{ahit}^{0}\leq N_{a1}^{\max}I_{aht}^{0}$$

(A27) $$F_{aht}^{0}\leq N_{a2}^{\max}I_{aht}^{0}$$

(A28) $$G_{aht}^{0}\leq N_{a3}^{\max}I_{aht}^{0}$$

Constraints (A26)–(A28) ensure the capacity constraint for the number of components shipped from the disassembly centers to the refurbished centers, recycle centers and disposal centers, i.e., that the number of components obtained at the disassembly centers and shipped to the refurbishing centers, recycle centers and disposal centers are kept within the specified maximum proportions, respectively.

Impact index constraints

(A29) $$K_{3}=\sum_{t}I_{t}-\frac{\sum_{t}J_{t}}{\sum_{t}I_{t}}$$

(A30) $$K_{4}=\frac{\sum_{a}\sum_{h}\sum_{i}\sum_{t}F_{ahit}^{0}}{\sum_{a}\sum_{b}\sum_{c}\sum_{t}B_{abct}^{0}}+\frac{\sum_{c}\sum_{t}\left(L_{ct}^{0}+J_{ct}^{0}\right)}{\sum_{c}\sum_{t}K_{ct}^{0}}$$

Constraints (A29) and (A30) calculate the impact index of social sustainability criteria $C_{3}$ and $C_{4}$, respectively. By referring to the concept proposed by Govindan et al. [46], the investment of $C_{3}$ is directly proportional to the revenue of the entire system, so the calculation of the impact index for $C_{3}$ depends on the revenue and cost. They also proposed that the value of $C_{4}$ is directly proportional to component and product recovery, so the calculation of the impact index for $C_{4}$ depends on the number of products produced and reused, as well as the number of components produced and reused.

(A31) $$\sum_{w}A_{w}^{0}\geq 1,w=b,c,d,e,g,h,i$$

Constraints (A31) ensure that at least one CPU, factory, warehouse, retailer, and collection, disassembly and refurbishing center is constructed. The importance of these constraints is that, if the number of an entity is 0, the network structure studied is incomplete and the corresponding supply chain activities cannot be performed. Therefore, it is essential that the sum of the decision variables related to the entity construction is not less than one.

Binary variable constraints and nonnegative constraints

(A32) $$\forall b,c,d,e,g,h,i,A_{w}^{0}\in \left\{0,1\right\},w=b,c,d,e,g,h,i$$

(A33) $$\begin{array}{l} B_{abct}^{0},B_{cdt}^{0},C_{det}^{0},C_{dft}^{0},C_{eft}^{0},D_{fet}^{0},D_{kfet}^{0},D_{fgt}^{0},D_{kfgt}^{0},D_{egt}^{0},D_{kegt}^{0},\\ E_{gct}^{0},E_{ght}^{0},F_{ahit}^{0},F_{aht}^{0},G_{aht}^{0},G_{aict}^{0},H_{dt}^{0},H_{d\left(t-1\right)}^{0},I_{aht}^{0},J_{ct}^{0},J_{jct}^{0},K_{ct}^{0},K_{jct}^{0},\\ L_{ct}^{0},L_{jct}^{0}\geq 0 \end{array}$$

Constraints (A32) and (A33) are binary variable constraints and nonnegative constraints associated with decision variables, respectively.

## Appendix C

**Theorem** **1.**
*There is at least one Pareto optimal solution in a given non-empty solution set.*

**Proof** **1.** Suppose that there are *n* elements in any solution set, a solution set containing *n* elements is obtained when solving the optimization problem of minimizing *n*-dimensional vector. Suppose that solving a one-dimensional equation yields *n* one-dimensional vectors. Sort these vectors from small to large so that a sorted vector by definition can be obtained. Consider two cases below. First, the smallest vector is strictly less than or equal to all other vectors. Since all vectors are hierarchical, this vector is a Pareto dominant vector and the solution it determines is the Pareto optimal solution according to the definition. Second, starting with the smallest vector, the values of two or more vectors are on an equal footing. Since the vectors are sorted out, it can be determined that these vectors are non-inferior because no other vectors are better than them, and there is no mutual dominance. Therefore, since they are non-inferior vectors, their corresponding solutions are also Pareto optimal solutions. The above proof is generalized to the general case, and when the vector is extended to two or *n* dimensions, the reasoning is also true. Unless the sorting is only done on a partial vector, it does not necessarily hold. If the original problem is to find the maximum value, the order of sorting is changed from maximum to minimum. As can be seen from the above proof process, any non-empty solution set contains at least one Pareto optimal solution. □

**Theorem** **2.**
*For a sufficiently small ζ>0, if f(x) is continuous over the search space [L,U], then the ε-constraint method converges with probability 1 to the optimal solution set with ζ precision, i.e.,*

(A34) $$P\left\{\underset{t\to \infty }{\lim}∥p\left(t\right)-p_{0}{∥}_{\infty }\leq \zeta \right\}=1$$

*where $p_{0}$ is the Pareto optimal solution set, $∥p\left(t\right)-p_{0}{∥}_{\infty }\leq \zeta$, means $\left\{∥x-x^{*}{∥}_{\infty }\leq \zeta |\forall x\in p\left(t\right),\exists x^{*}\in p_{0}\right\}$.*

**Proof** **2.** First prove that for any two points $x^{\prime }$ and x, $x^{\prime },x\in \left[L,U\right]$, $x^{\prime }$ is accessible with ζ precision from x through hybridization and mutation of the ε-constraint method, i.e.,

(A35) $$P\left\{∥MC\left(x\right)-x^{\prime }{∥}_{\infty }\leq \zeta \right\}>0$$

where MC(x) is the point produced from x by hybridization and mutation operators. In fact, let $\bar{x}$ be any point generated from x by the hybridization operator, that is, $C(x)=\bar{x}$, as long as proof $x^{\prime }$ is accessible with ζ precision from $\bar{x}$, that is, it satisfies the following inequalities.

(A36) $$P\left\{∥MC\left(\bar{x}\right)-x^{\prime }{∥}_{\infty }\leq \zeta \right\}>0$$

In fact, if for any component ${x^{\prime }}_{i}$ of $x^{\prime }$, let ${x^{\prime }}_{i}\in \left[\omega_{k_{i}}^{i},\omega_{k_{i}+1}^{i}\right]$, and the probability that the mutation operator randomly selects the natural number $k_{i}$ in $\left\{1,2,\ldots ,n_{i}-1\right\}$ is $\frac{1}{n_{i}-1}$. According to the variation method, it can be known that ${\bar{x}}_{i}^{t}\in \left[\omega_{k_{i}}^{i},\omega_{k_{i}+1}^{i}\right]$ is obtained by mutating the *i*th component $x_{i}^{t}$ of $\bar{x}$, then $|x_{i}^{t}-{\bar{x}}_{i}^{t}|\leq \zeta$ must be true, so the probability that the *i*th component of $\bar{x}$ mutation satisfies $|x_{i}^{t}-{\bar{x}}_{i}^{t}|\leq \zeta$ is $\frac{1}{n_{i}-1}>0$. In addition, because they are independent when mutating, then the following formula can be satisfied.

(A37) $$P\left\{∥MC(\bar{x})-x^{\prime }{∥}_{\infty }\leq \zeta \right\}=\frac{1}{n_{1}-1}•\frac{1}{n_{2}-1}\ldots \frac{1}{n_{n}-1}=\prod_{i=1}^{n}\frac{1}{n_{i}-1}>0$$

Therefore, $x^{\prime }$ is accessible with ζ precision from x through hybridization and mutation of the ε-constraint method. The sequence generated by the ε-constraint method is ∀(t), any solution in p(t+1) is not inferior to the solution in p(t), or improves the solution in p(t) (at least not worse than the solution in p(t)), so p(1),p(2),…p(t),… is monotonic, then the formula $P\left\{\underset{t\to \infty }{\lim}∥p(t)-p_{0}{∥}_{\infty }\leq \zeta \right\}=1$ is monotonic, then the formula can be satisfied, namely, the ε-constraint method converges with probability 1 to the global optimal solution set of the problem. □
